# Electrophysiologically calibrated optogenetic stimulation of dentate granule cells mitigates dendritic spine loss in denervated organotypic entorhino-hippocampal slice cultures

**DOI:** 10.1038/s41598-025-88536-w

**Published:** 2025-02-07

**Authors:** Tijana Hanauske, Carolin Christina Koretz, Tassilo Jungenitz, Jochen Roeper, Alexander Drakew, Thomas Deller

**Affiliations:** 1 Institute for Clinical Neuroanatomy, Dr. Senckenberg Anatomy, Faculty of Medicine, Goethe University Frankfurt, Theodor-Stern-Kai 7, 60590 Frankfurt, Germany; 2 Institute for Neurophysiology, Faculty of Medicine, Goethe University Frankfurt, Theodor-Stern-Kai 7, 60590 Frankfurt, Germany

**Keywords:** Biological techniques, Cell biology, Neuroscience, Structural biology, Anatomy

## Abstract

Organotypic slice cultures (OTCs) are versatile tools for studying long-term structure-function relationships of neurons within a defined network (e.g. hippocampus). We developed a method for repeated experimenter-controlled activation of hippocampal granule cells (GCs) in OTCs within the incubator. After several days of contact-free photonic stimulation, we were able to ameliorate entorhinal denervation-induced structural damage in GCs. To achieve this outcome, we had to calibrate the intensity and duration of optogenetic (light) pulses using whole-cell electrophysiological recordings and multi-cell calcium imaging. Our findings showed that ChR2-expressing cells generated action potentials (APs) or calcium transients in response to illumination but were otherwise functionally indistinguishable from non-transduced GCs within the same neural circuit. However, the threshold for AP firing in single GCs varied based on the stimulus light intensity and the expression levels of ChR2. This information allowed us to calibrate light intensity for chronic stimulations. Denervated GCs exhibited significant spine loss four days post-denervation, but this detrimental effect was mitigated when AP firing was induced at a physiological GC bursting rate. Phototoxic damage caused by chronic light exposure was significantly reduced if illuminated with longer wavelength and by adding antioxidants to the culture medium. Our study presents a versatile approach for concurrent non-invasive manipulation and observation of neural circuit activity and remodeling in vitro.

## Introduction

Organotypic slice cultures (OTCs) of the hippocampal formation are an established in vitro model to study neuronal activity, connectivity, and structure under physiological and pathological conditions^1–4^. The OTCs exhibit the characteristic network of principal hippocampal neurons and glial cells and maintain the basic synaptic connectivity of the hippocampus^5–7^. Furthermore, OTCs mature in vitro, making it possible to study developmental changes of identified neural cells^8–11^.

OTCs do not receive extrinsic afferent input. Nevertheless, they generate spontaneous network activity which propagates throughout the OTC circuit^12,13^. Using pathway stimulation^14,15^, treatment with TTX^16,17^ as well as entorhinal denervation^18^, a wide range of induced plasticity phenomena has previously been investigated in OTCs. Using these tools, mechanisms of Hebbian plasticity^15,19^, homeostatic plasticity^17,20^, denervation-induced plasticity^21–24^ as well as metaplasticity^17^ were identified.

A limitation of the above approaches is the use of stimulation and/or recording electrodes requiring physical tissue contact. Typically, such cultures are subsequently fixed or discarded after the experiments to avoid tissue contamination. In contrast, stimulation of OTCs with light allows for a contact-free activation of optogenetically transduced neurons^25^ and may enable a cell-specific “read-in” of complex activity patterns into OTCs over extended time periods, i.e. days or even weeks. Furthermore, this could be combined with an optical read-out using genetically encoded calcium sensors for a contact-free all-optical approach. To demonstrate the feasibility of such an approach, we used local injections of an adeno-associated viral vector^11,23,26^ to selectively transduce mouse GCs with a Channelrhodopsin-2 (ChR2)-tdTomato construct^27–29^. Patch-clamp recordings were employed to verify that basic properties of GCs were not affected by transgene expression and to demonstrate suprathreshold activation of transduced GCs by light illumination. Activation of GCs was also monitored using a genetically encoded calcium indicator^30^ which revealed robust calcium transients within transduced neurons. Finally, we tested whether direct optogenetic activation of GCs can compensate for denervation-induced loss of synaptic activation. For this, we cut the perforant path, thus removing ~ 80% of excitatory synapses of GCs^3,31,32^. Whereas denervated and non-illuminated GCs exhibited a characteristic denervation-induced spine loss four days after denervation^32^, spine loss was mitigated in denervated and illuminated GCs.

## Materials and methods

### Animals/ethics statement

The generation of organotypic entorhino-hippocampal slice cultures from C57Bl6/J mice was performed in accordance with the German animal welfare law (TierSchG) and was approved by the Animal Welfare Officer of Goethe-University, Faculty of Medicine (Wa-2014-35). Mice were bred and housed at the animal facility of the Goethe-University Hospital Frankfurt, and were maintained on a 12 h light/dark cycle with food and water available *ad libitum*. Every effort was made to minimize distress and pain of animals. Methods are reported in accordance with the Animal Research: Reporting of In Vivo Experiments (ARRIVE) guidelines.

### Organotypic slice culture preparation

Organotypic entorhino-hippocampal slice cultures (OTCs) were prepared from C57BL/6 mice of both sexes at postnatal days 4–5 as described previously^33^. In brief, mice were sacrificed via rapid decapitation and 300 μm thick horizontal brain sections were prepared using a vibratome (LeicaVT1200S). The hippocampi with entorhinal cortices were dissected out and placed onto sterile membrane culture inserts (Millipore Millicell-CM, PICM ORG 50, 0.4 μm pore size, 30 mm diameter). OTCs were cultivated in a humidified incubator (95% air, 5% CO_2_, at 35 °C) with incubation medium containing 42% MEM, 25% Basal Eagle Medium containing Earle’s salts, 25% heat-inactivated normal horse serum, 0.65% glucose, 25 mM HEPES, 0.1 mg/ml streptomycin, 100 U/ml penicillin, 0.15% sodium bicarbonate, and 2 mM glutamax (pH 7.30). The incubation medium was changed every 2 to 3 days until further processing. Since cultures continue to mature in vitro^8–11^, OTCs were incubated for 21–30 days to ensure full maturation and a strong expression of virally transduced constructs. Only healthy cultures without signs of structural alteration were included in the experiments and were randomly assigned to control or experimental groups.

### Adeno-associated viral vector transduction in OTCs

For transduction of the ChR2 into living neurons, we used adeno-associated viral (AAV) vectors that contained sequences of the ChR2-H134R fused with either enhanced yellow fluorescent protein (EYFP) under the CamKIIα promoter or tdTomato under the neuronal Synapsin1 promoter. Shortly after the preparation of entorhino-hippocampal OTCs, dentate granule cells (GCs) were transduced with AAV2-CamKIIα-ChR2-EYFP (AddGene #26969) diluted to a viral titer of 2 × 10^12^ vg/ml in PBS or AAV2-hSyn1-ChR2-tdTomato (Vector Builder) via local injection into the dentate gyrus (DG) at day in vitro 2 (DIV 2). Injections were essentially performed as follows: Each membrane insert containing the OTCs was placed in a 30 mm petri dish lid containing 1 ml pre-warmed (37 °C) “Modified Edi’s Recording Medium” (MERM) which consisted of 129 mM NaCl, 4 mM KCl, 1 mM MgCl_2_, 2 mM CaCl_2_, 4.2 mM glucose, 10 mM HEPES, 0.1 mM Trolox, 0.1 mg/ml streptomycin, 100 U/ml penicillin, with a pH of 7.40. The osmolarity was adjusted to match the incubation medium. The OTCs were covered with 1.5 ml MERM and placed onto the stage of an upright microscope (Nikon Eclipse FN1) that was equipped with a camera (Tucsen Photonics TrueChrome Metrics) and a 10 × (0.3 NA, dipping; Nikon) objective. Injection pipettes (3-3.5 MΩ when filled with MERM) were produced using a micropipette puller (DMZ universal puller; Zeitz) to pull glass capillaries with filament (Harvard apparatus GC150TF-10) so that a narrow tip was formed. Injection pipettes were loaded with 3 µl viral solution and placed in the microJECT pipette holder (npi electronic) which was attached to a micromanipulator (Luigs & Neumann SM-5). Pressure pulses were delivered with a pressure application system (npi electronic PDES-DXH). The injection pipette was positioned just below the granule cell layer (GCL) of the DG and the viral solution was injected with a pressure of 0.04 bar for 5 s, thereby distributing the viral solution within the suprapyramidal blade of the GCL. Transduced dentate GCs expressed ChR2 throughout the entire cell membrane, thus enabling the visualization of the complete morphology of each neuron. For calcium imaging experiments, OTCs were transduced with the genetically encoded calcium indicator jRCaMP1b^30^ via bulk loading of 0.7 µl of AAV9-Syn-NES-JRCaMP1b (Addgene #100851), diluted in PBS to a titer of 4 × 10^12^ vg/ml.

### Optogenetic stimulation of dentate granule cells in OTCs

Dentate GCs that had been transduced with ChR2-H134R were stimulated using blue (Royal-Blue (447.5 nm) LUXEON Rebel LED Part No. LXML-PR02-A900) or green LEDs (Cyan (505 nm) LUXEON Rebel LED Part No. LXML-PE01-0070) which were part of modules of three LEDs soldered to a thermal sink pad (Royal-Blue: LUXEON Part No SR-03-RA900, Cyan: LUXEON Part No SP-03-C2). LED modules of the same type were mounted on a passive aluminum cooler and covered with circular collimators to allow simultaneous stimulation of multiple OTCs placed in a 6-well-plate. LEDs were powered with an LED driver and controlled via a Raspberry Pi computer, using an arrangement similar to the one employed by Wefelmeyer et al. (2015). The light power of the LEDs was measured with a power meter (Thorlabs, PM160T) at a distance of 2 cm from the LED at successive increments relative to the maximum drive current. The measured optical power was then divided by the sensor size to obtain the light output in mW/mm². For better comparability of the light intensities, the LEDs were always positioned approximately 2 cm away from the cultures during electrophysiological experiments, calcium imaging, and chronic photostimulation in the incubator. During electrophysiological recordings, a single LED set was mounted on a micromanipulator and positioned opposite to the recording pipette. To ensure optimal illumination of the culture, the LED was positioned while measuring the stray light of the LED through the objective lens using one of the confocal PMT detectors. Trains of five 10 ms light pulses at an inter-pulse-interval of 2 s were applied while recording the activity of individual ChR2-expressing GCs using whole-cell patch-clamp recordings. For calcium imaging experiments, one LED set was placed directly underneath the glass bottom of the imaging chamber replacing in position the substage condenser. Cultures were stimulated with three times 5 pulses (500 ms pulse length) at 1 Hz with a light intensity of ~ 8.0 mW/mm². For chronic photostimulations, six-well plates containing the OTC filters were placed inside a stimulation box containing an LED assembly of six LED sets, one set per culture insert. In experiments examining the extent of cell death after chronic light stimulation using propidium iodide (PI), 100 light pulses (10 ms) at 12.5 Hz once per minute were delivered with a light intensity of ~ 4.5 mW/mm² for 505 nm LEDs and ~ 4.0 mW/mm² for 450 nm LEDs. For chronic photostimulation over four days, 450 nm light pulses (10 ms) at ~ 4.0 mW/mm² were delivered in trains of five at 12.5 Hz every hour for 90 h. In experiments using antioxidants to protect cultures from potential phototoxic effects, the following were applied directly to the incubation medium: 3.2 µM glutathione, 10 nM catalase, 110 µM ascorbic acid, 100 µM trolox, 2.3 µM α-tocopherol, 77 nM superoxide dismutase (all from Sigma-Aldrich; see Grubb & Burrone, 2010).

### Whole-cell patch-clamp recordings

Whole-cell patch-clamp recordings were performed 25–30 days post viral transduction. Cultures were transferred to a custom-made, temperature-controlled recording chamber mounted on the stage of a confocal laser scanning microscope (Fluoview 3000 on a BX63L upright microscope stand, equipped with a 60x NA 1.0 water immersion objective lens, Olympus/Evident) where they were superfused with artificial cerebrospinal fluid (ACSF) containing 126 mM NaCl, 2.5 mM KCl, 1.25 mM NaH_2_PO_4_, 2 mM MgCl_2_, 2 mM CaCl_2_, 26 mM NaHCO_3_ and 10 mM glucose, oxygenated with carbogen (95% O_2_/ 5% CO_2_) at 35 °C. Excitatory and inhibitory synaptic transmission was blocked by 10 µM CNQX (6-cyano-7-nitroquinoxaline-2,3-dione, Alomone labs), 10 µM D-AP5 (D-2-amino-5-phosphonopentanoic acid, Alomone labs) and 10 µM gabazine (SR95531 hydrobromide, Alomone labs). 3–7 MΩ patch-pipettes were pulled from borosilicate glass capillaries (Harvard Apparatus GC150TF-10) with a DMZ universal puller (Zeitz) and filled with K-gluconate-based internal solution: 126 mM K-gluconate, 4 mM KCl, 10 mM HEPES, 2 mM MgATP, 0.2 mM NaGTP, 10 mM phosphocreatine, and 0.3% (w/v) biocytin, pH 7.25, osmolarity adjusted to 290–300 mOsm/kg with sucrose. 10 µM Alexa 488 hydrazide was added to the internal solution to immediately visualize the recorded cell. Dentate GCs were identified by IR-Dodt gradient contrast (Luigs & Neumann) and ChR2 expression was confirmed with a short scan using a 561 nm laser. Signals were recorded using either an ELC-03X amplifier (npi electronics), digitized using a PXI multifunctional I/O-module (NI PXI-6259 card in a NI PXI-1033 chassis, National Instruments) at a rate of 100 kHz and visualized with an in-house software written in LabView (version 2023, National Instruments), or a Multiclamp 700 B amplifier, Digidata Digitizer, pClamp Software (Molecular Devices). Recordings obtained with both systems were pooled. To determine the light intensity necessary to elicit an AP, cells were recorded in current-clamp mode and optically stimulated with increasing light intensities from 10 to 100% of the maximum LED drive current (1000 mA) in 10% increments. For every increment, the cells were stimulated with 10 light pulses (each pulse: 10 ms). Passive and active properties were measured by injecting 1 s square pulse currents starting at − 100 pA and increasing in 10 pA steps up to 300 pA (sweep duration: 2 s). The resting membrane potential was determined as the median potential of the cell before onset of each of the first 20 stimulus current steps. The input resistance was determined as the median of the ratios of the first 20 decaying voltage steps after the end of each stimulus current by the amplitude of that current step. Membrane time constant was obtained as the median time to decay to 1/e of the voltage amplitude of the same voltage steps fitted to single exponential decays. Cell capacitance was calculated as the ratio of membrane time constant by input resistance for each step. The threshold potential of the first AP elicited in each cell was determined as the break-off point of its phase plot (dV/dt versus V). The rheobase was the current step amplitude eliciting that very first AP. During optical stimulation, the membrane potential values of the cells were recorded with no current injected. After a successful recording, a confocal image stack of the recorded cell was acquired (FV3000, 488 nm and 561 nm excitation lasers; 1024 × 1024 pixel; zoom 1.8; voxel size 0.23 × 0.23 × 1 μm).

### Calcium imaging

Cultures were locally injected with the AAV2-CamKIIa-ChR2-EYFP in the GCL as described above. Additionally, the cultures were transduced with the genetically encoded calcium indicator jRCaMP1b^30^ via bulk loading of 0.7 µl of AAV9-Syn-NES-JRCaMP1b, diluted in PBS to a titer of 4 × 10^12^ vg/ml, onto the whole OTC. Imaging experiments were performed 28 days after viral transduction. Slice cultures were cut out of the membrane insert and placed into an imaging chamber (Luigs & Neumann). Imaging was performed at RT under continuous perfusion with oxygenated ACSF. Time-lapse image series with a resolution of 512 × 512 were recorded with a frame rate of 4 fps using an LSM 5 Pascal confocal microscope (Zeiss) and a 40x water immersion objective (0.8 NA; Zeiss) with the pinhole fully opened to 2.0 airy units. Baseline fluorescence was recorded for at least 20 s before the stimulation was started (for detailed stimulation protocol, see above). A reference image taken of the ChR2-EYFP expression was used to manually select the regions of interest (ROI) for the analysis. Fluorescence signals from the selected ROIs were normalized by subtracting the background fluorescence and dividing it by the baseline fluorescence. To assess signal-to-noise ratio (SNR), the baseline fluorescence over the first 30 s before the stimulation was used to calculate the noise level. It was defined as the standard deviation of signals across all cells during this 30 s baseline window. The peak response for each cell was divided by the noise level to obtain the SNR. Based on their SNR values, cells were categorized into two groups: GCs exhibiting calcium transients (SNR > 20) and GCs not exhibiting calcium transients (SNR < 20).

### Propidium iodide staining

To assess phototoxic damage, OTCs were incubated with propidium iodide (PI, 5 µg/ml) for 2 h following chronic photostimulation. PI is a nucleic acid intercalating dye that is only permeant to damaged or dead cells^34^. Subsequently, OTCs were washed with PBS and fixed with 4% PFA/4% sucrose in PBS for 1 h and 2% PFA/30% sucrose overnight. Following fixation, OTCs were washed with PBS and stained with the nuclear marker TOPRO-3 (1:30,000 in PBS) for 10 min. After two additional washes with PBS, OTCs were mounted onto glass slides using Dako fluorescence mounting medium.

### Confocal imaging

Confocal image stacks of fixed OTCs were acquired with a laser scanning microscope (Nikon C2si; 561 nm and 640 nm excitation lasers) equipped with two GaAsP detectors using the NIS-Elements software at a resolution of 1024 × 1024 pixels. Image stacks (step size 5 μm) were obtained using a 4 × (0.2 NA; Nikon) or a 10 × (0.3 NA) objective. Live cell imaging of ChR2-tdTomato-labeled GC dendritic spines was performed 23–28 days following viral transduction in MERM buffer with a pH of 7.40. The osmolarity of the imaging medium was adjusted with sucrose to match the osmolarity of the incubation medium. High resolution image stacks (1024 × 1024 pixels, 2x average per frame) of dendritic GC segments located in the outer molecular layer were recorded with a 60x water-immersion objective (1.0 NA), applying a 5x field zoom and a z-axis interval between consecutive frames of 0.30 μm. The pinhole opening was set to 1.0 airy units. The same dendritic segments were imaged right before optogenetic stimulation (day 0) and following 90 h of chronic photostimulation (day 4; for detailed stimulation protocol, see above).

Some cultures were imaged with a Fluoview 3000 confocal microscope (561 nm excitation laser) equipped with two GaAsP detectors using the FV31S-SW software. Individual membrane inserts containing the OTCs were placed onto a custom-made temperature- and CO_2_-regulated imaging chamber. During imaging, OTCs were retained in incubation medium (pH 7.30) that was constantly oxygenated with carbogen gas (95% O_2_, 5% CO_2_). A continuous flow of dH_2_O into the imaging chamber ensured a constant osmolarity value of the incubation medium, compensating for evaporation from the heated medium. Image stacks (512 × 512 pixels, 4x average per frame) were obtained with a 60 × (1.0 NA; Olympus) water immersion objective lens, a 5x field zoom and a z-axis interval of 0.25 μm between consecutive frames. The pinhole opening was set to 1.0 airy units. Again, the same dendritic segments were recorded at day 0 right before optogenetic stimulation as well as after four days of photostimulation (for detailed stimulation protocol, see above). Data obtained with the two live imaging setups were comparable and thus the datasets were pooled.

### Perforant Path Lesion

Cultures were allowed to mature for 30–32 DIV. Using a sterile scalpel blade, the entorhinal cortex was cut away from the culture and removed from the culture dish, as previously described^3,11,22,23^. After the lesion, cultures were placed back into the incubator and maintained for four days post lesion. Time-matched control cultures were treated in the same way but did not receive a lesion.

To study the effect of chronic light activation on denervated GCs, two conditions with two groups of time-matched cultures were compared. Non-illuminated ChR2-tdTomato cultures served as controls: Within this cohort, one group of cultures was non-denervated and the second group of cultures was denervated. Illuminated ChR2-tdTomato cultures were the experimental group: Within this cohort, one group of cultures was non-denervated and the second group of cultures was denervated.

### Histological data analysis

All analyses of histological data were performed using ImageJ (version 1.52 h^35^) as part of the Fiji distribution package^36^.

The level of ChR2 expression was analyzed in neurons of which confocal image stacks were available following patch-clamp experiments. ChR2-tdTomato-related fluorescence in the cell membrane of GC somata was measured in the frame where the largest portion of the membrane was imaged vertically as identified by a linear appearance of fluorescence. A ROI encompassing the narrow linear part of the ChR2-tdTomato-labeled cell membrane delineating the GC soma was created using the polygon selection tool. The observed fluorescence within such defined ROIs results from the convolution of the point spread function with the vertically oriented membrane. Since the membrane and therefore the fluorescent dye represents a 2D-sheet, the total fluorescence intensity obtained within each ROI was divided by the length of the ROI representing the length of membrane section. This value is a measure of the density of fluorescent ChR2-tdTomato channels per unit membrane area.

To evaluate the extent of PI expression following prolonged high-frequency photostimulation, each confocal image stack was first transformed into a z-projection and then converted into a binary image. A threshold was applied to divide all pixels into either foreground or background signal. The threshold value was set by using a preview of the image histogram to ensure that the remaining fluorescence signal corresponded mainly to cell nuclei, i.e. small cell-sized patches of fluorescence. The number and distribution of pixels was calculated using the histogram function. The number of pixels representing the foreground PI signal within the image was divided by the total number of pixels to obtain a normalized fluorescence value (fraction of area) corresponding to the density of PI-positive cells.

For analysis of changes in GC spine density following chronic photostimulation, confocal image stacks of ChR2-tdTomato-labeled GC spines were deconvolved using Huygens Professional Version 17.10 (Scientific Volume Imaging, The Netherlands, http://svi.nl). Only images showing sufficient resolution for analysis were included. Image processing for brightness and contrast and data analysis were performed using ImageJ (version 1.52 h^35^). 1–3 dendritic segments per culture were imaged. Each dendritic segment was measured using a segmented line tracing tool and mature dendritic spines displaying a clear head^37^ were selected using the custom-made “SpineAnalyzerJ” plugin for ImageJ. The number of spines was determined and spine density per µm segment length was calculated. The experimenter (TH) was blinded to imaging time point and treatment condition during analysis. Relative changes in spine density on day 4 relative to day 0 were compared in time-matched non-denervated and denervated OTCs that were either non-illuminated (control condition) or illuminated (experimental condition).

### Statistical analysis

We conducted two different experiments sharing a very similar design: In both cases we aimed to elucidate the interaction of two different treatments (categorial factors) on one target parameter (a numerical value).  In one experiment, we analyzed the effects of entorhinal lesion (“Treatment”: “ECL” and “NoECL”) and of optogenetic stimulation (“Stimulation”: “LED” and “NoLED”) on the change of spine density of dendritic segments from day 0 to day 4. The other experiment assessed the effects of optogenetic stimulation using two different wavelengths (“Stimulation”: “LED450”, “LED505”, and “NoLED”) and the application of antioxidants (“Antioxidants”: “AO” and “NoAO”) on the amount of cell death (quantitative measure of Propidium Iodide staining). Therefore, a robust ANOVA allowing for unbalanced design, non-normality, and inhomogeneous variances involving two fixed crossed factors was employed. We used the function “GFD” of the R-package “GFD” (version 0.3.3^38^, R Statistical Software: vR-4.4.1^39^). Following the recommendations in Friedrich et al. (2017), the Wald-type statistic “WTS” was considered, and the P-values were obtained by permutation of the data among groups (“nperm = 100.000”). This was followed by pairwise *post-hoc* comparisons of reasonable combinations of treatment groups using two-sample Brunner-Munzel tests (function “brunnermunzel.permutation.test” of the R-package “brunnermunzel”, test statistic “B”, P-values obtained by exhaustive permutation). This nonparametric test is a less constraint substitute for the common “U-test” having more power and allowing for inhomogeneous variances between samples^40^.

Statistical comparison of electrophysiological parameters between ChR2-tdTomato-positive and ChR2-tdTomato-negative cells was employed using the two-sample Brunner-Munzel test.

Graphs were generated using GraphPad Prism v8.4. Significance level was set to *P* < 0.05.

## Results

### A subgroup of GCs expresses ChR2-tdTomato after targeted injection of the viral vector into the GCL

**Fig. 1:**
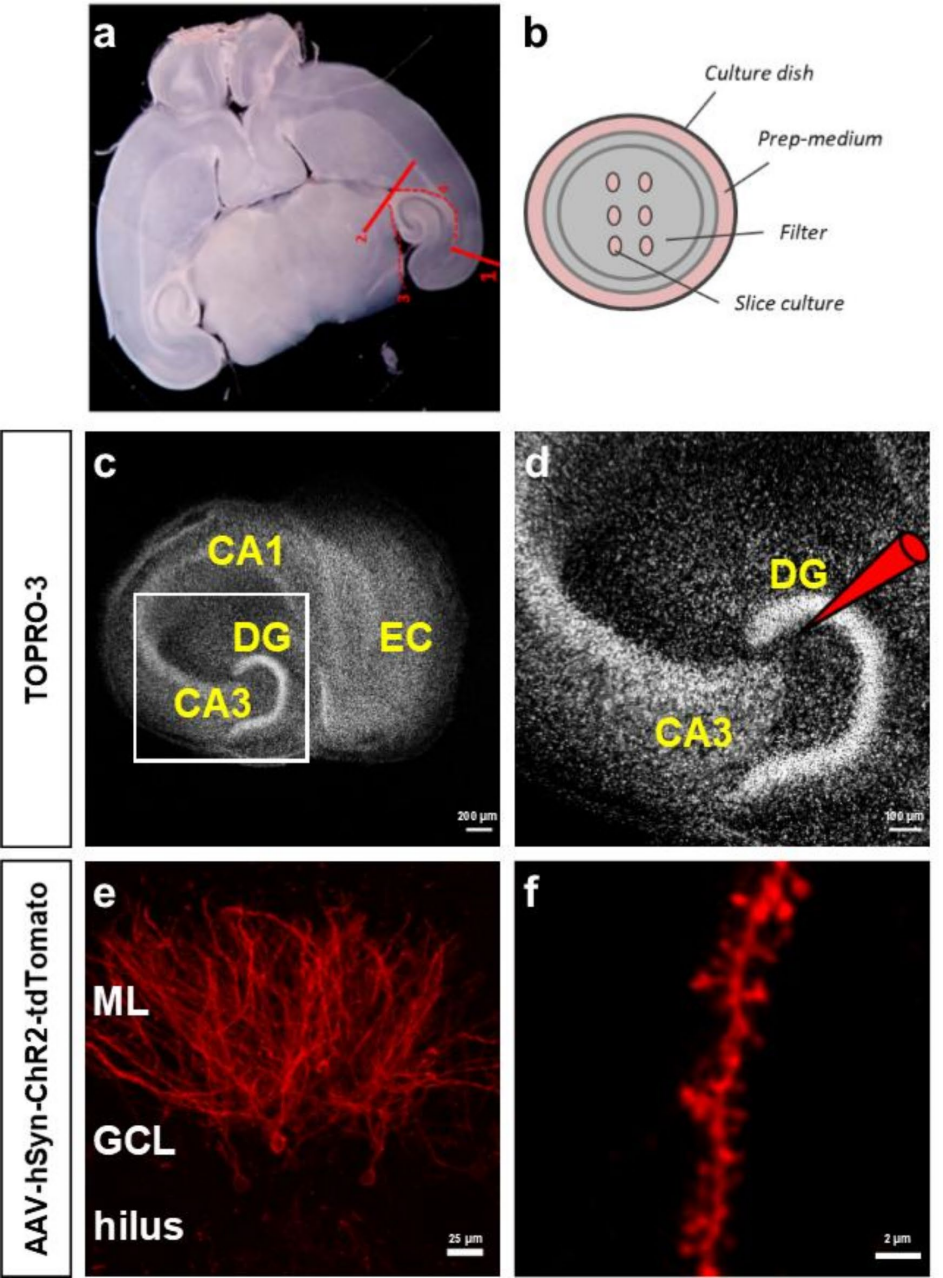
A subgroup of GCs expresses ChR2-tdTomato after targeted injection of the viral vector into the GCL. (**a**) OTCs were prepared from horizontal brain sections from which the hippocampus with part of the entorhinal cortex (EC) were dissected out. (**b**) The cultivation of OTCs was done on porous membrane inserts in a controlled environment, so that they were easily accessible and could stay viable for extended periods of time. (**c**) OTCs contain a preserved entorhino-hippocampal neuronal network, including part of the EC, the dentate gyrus (DG) and the Cornu Ammonis (CA) areas. (**d**) Local injection of an adeno-associated viral (AAV) vector containing ChR2-tdTomato into the DG led to specific transduction of a subset of dentate granule cells (GCs) throughout their entire structure, including apical dendrites that extended into the molecular layer (ML) of the DG (**e**). (**f**) Viral transduction with ChR2-tdTomato enabled labeling of fine morphological structures, including dendritic spines. GCL: granule cell layer.

Complex organotypic slice cultures (OTCs) consisting of entorhinal cortex and hippocampus were prepared by dissecting the entorhinal cortex and hippocampus out of horizontal mouse brain slices (Fig. 1a). Because the entorhinal cortex remains attached to the hippocampus, the entorhinal afferents re-establish connections with their hippocampal targets^41^. Several OTCs were cultivated on one semi-porous filter membrane insert and arranged systematically on the membrane (Fig. 1b). An adeno-associated viral (AAV) vector was pressure-injected into or near the suprapyramidal blade of the GCL under visual control (Fig. 1c, d).

Expression of ChR2-tdTomato began around 10 days and was strong from 28 days post injection (dpi) on. Cellular expression levels varied between single transduced GCs, indicating that some GCs take up more virus and/or synthesized more protein than others. In strongly expressing GCs, ChR2-tdTomato labeled the entire neuron, i.e. the cell somata, dendritic processes extending into the molecular layer (ML) of the DG (Fig. 1e), as well as the axons projecting through the hilus to the CA3 region of the hippocampus. As ChR2-tdTomato expression is membrane-bound^28^, it offers visualization of all structures covered by the cell membrane, including fine morphological features such as dendritic spines with high resolution (Fig. 1f), allowing for a detailed structural analysis of the transduced neurons.

### Optogenetic activation of ChR2-expressing dentate GCs in OTCs depends on light intensity and cellular ChR2-expression levels

After successful transduction of the ChR2-H134R into dentate GCs, we tested whether ChR2-expressing GCs could be activated with light. Effects of stimulation wavelength and light intensity were studied, as well as the effect of the expression level of ChR2 in identified GCs.

**Fig. 2:**
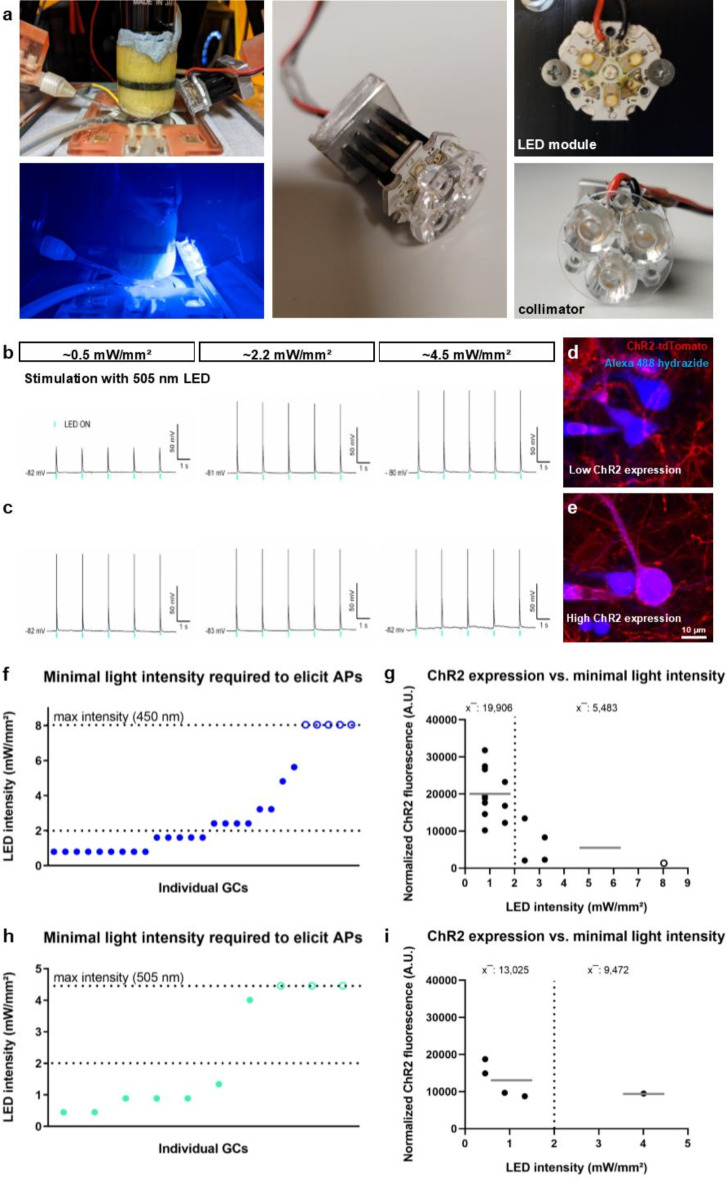
Optogenetic activation of ChR2-expressing dentate GCs in OTCs depends on light intensity and cellular ChR2-expression levels. (**a**) Patch-clamp recordings of single ChR2-tdTomato expressing GCs were performed while cells were stimulated with either blue (450 nm) or green (505 nm) light. Photostimulation was carried out with high power LED modules consisting of 3 LEDs mounted onto a custom-made fixture and covered with a collimator. (**b**, **c**) Current-clamp recordings were performed while GCs were stimulated with 450 nm or 505 nm light pulses (10 ms) at different intensities. Sample traces of stimulations with 505 nm LEDs: While some cells frequently exhibited subthreshold depolarization in response to light pulses at ~ 0.5 mW/mm² LED intensity (b), others reliably fired APs in response to light pulses at all levels of intensity (**c**). (**d**, **e**) Confocal image stacks of patched GCs filled with Alexa 488 visualize ChR2-tdTomato expression of the recorded neurons. (f) Analysis of the minimal light intensity required to prompt spiking revealed a proportion of neurons activated by relatively low light intensities (i.e. <2 mW/mm2; *n* = 14) while other neurons needed higher light intensities to spike (*n* = 8) or did not spike (open circles; *n* = 5). (**g**) An examination of ChR2 expression in individual GCs showed that cells responding to low light intensities exhibited a relatively high ChR2-related fluorescence density (gray line depicts mean: 19,906 A.U.; *n* = 11) compared with GCs that required higher light intensities or did not respond at all (mean: 5,483 A.U.; *n* = 5). (**h**) The majority of GCs that were stimulated with 505 nm LEDs fired APs in response to light intensities below 2 mW/mm2 (*n* = 6) while one neuron spiked at ~ 4.0 mW/mm2. Three GCs showed no response. (**i**) A ChR2 expression analysis showed that GCs that fired APs following stimulation with a low light intensity had higher expression levels (mean: 13,025 A.U.; *n* = 4) compared with the neuron that only responded to high light intensity (9,472 A.U.; *n* = 1). A.U.: arbitrary units. n represents number of cells.

First, patch-clamp recordings were performed to demonstrate light activation. For the ChR2 variant used here, the excitation peak is 450 nm^29^, although light of longer wavelength (e.g. 505 nm), which may be less phototoxic, is also expected to activate this ChR2 variant^29^. To verify and compare the two different stimuli, we used high power LED modules (Fig. 2a) with 450 nm as well as 505 nm wavelength for stimulation. As light intensity plays an important role in the activation of ChRs^29^, we recorded neuronal responses to different levels of LED intensity. Figure 2b, c display sample recordings of GC responses to 505 nm light. Some GCs showed depolarizing potential transients in response to each 505 nm light pulse at 10% LED intensity (~ 0.5 mW/mm^2^), but the voltage did not reach the threshold for spiking. However, at 50% (~ 2.2 mW/mm^2^) and 100% (~ 4.5 mW/mm^2^) light intensity, most of these neurons exhibited consistent firing of APs with each stimulating light pulse (Fig. 2b). On the other hand, another cohort of neurons fired APs in response to 505 nm light under all intensity conditions (Fig. 2c). Due to the variability in response rates between neurons, we determined the minimal light intensity that was required to elicit APs in individual GCs (Fig. 2f, h). Using the 450 nm LED, we found that a light intensity of up to ~ 2.0 mW/mm^2^ prompted APs in a subset of recorded neurons. However, some GCs required higher light intensities to reach the threshold for spiking. Five neurons did not fire any APs, even at the maximal available light intensity of ~ 8.0 mW/mm^2^ (Fig. 2f). We speculated that the differences in spiking response might be related to the expression level of ChR2-tdTomato (Fig. 2.d, e). Indeed, when we examined ChR2-tdTomato-related fluorescence density in GC somata, we found that GCs that were activated by relatively low light intensities (i.e. ~0.8 mW/mm^2^ and ~ 1.6 mW/mm^2^) exhibited relatively high ChR2 expression levels (mean: 19,906 A.U.). On the other hand, GCs that required higher levels of light intensity (i.e. ~2.4 and ~ 3.2 mW/mm^2^) to fire APs and one GC that did not spike at all, had a relatively low expression of ChR2 (mean: 5,483 A.U.; Fig. 2g). Stimulation trials with the 505 nm LED yielded comparable results. The majority of GCs fired APs at light intensities below ~ 2.0 mW/mm^2^, one neuron started firing only at ~ 4.0 mW/mm^2^, and three GCs did not fire at all, even at the maximal available light intensity (~ 4.5 mW/mm^2^; Fig. 2h). Again, GCs that fired APs at light intensities below ~ 2 mW/mm^2^ showed overall higher expression levels of ChR2 (13,025 A.U.) compared with one cell that required ~ 4.0 mW/mm^2^ to spike (9,472 A.U.; Fig. 2i). These findings suggest that the extent of ChR2 protein expression should be considered, as it may affect the response rate of individual neurons to a particular light stimulus.

### Optogenetic stimulation of ChR2-expressing GCs induces intracellular calcium transients

In a next step, we verified the robust activation of ChR2-transduced GCs by employing calcium imaging. The rationale behind this approach was - in addition to verifying the activation of GCs with a second technique - to demonstrate that GC activation can also be reliably read out of the cultures using light, i.e. by measuring fluorescence signals emitted from a genetic calcium indicator. Combining this approach with optogenetics in OTCs will allow to “read in” and to “read out” neuronal activity with light.

**Fig. 3:**
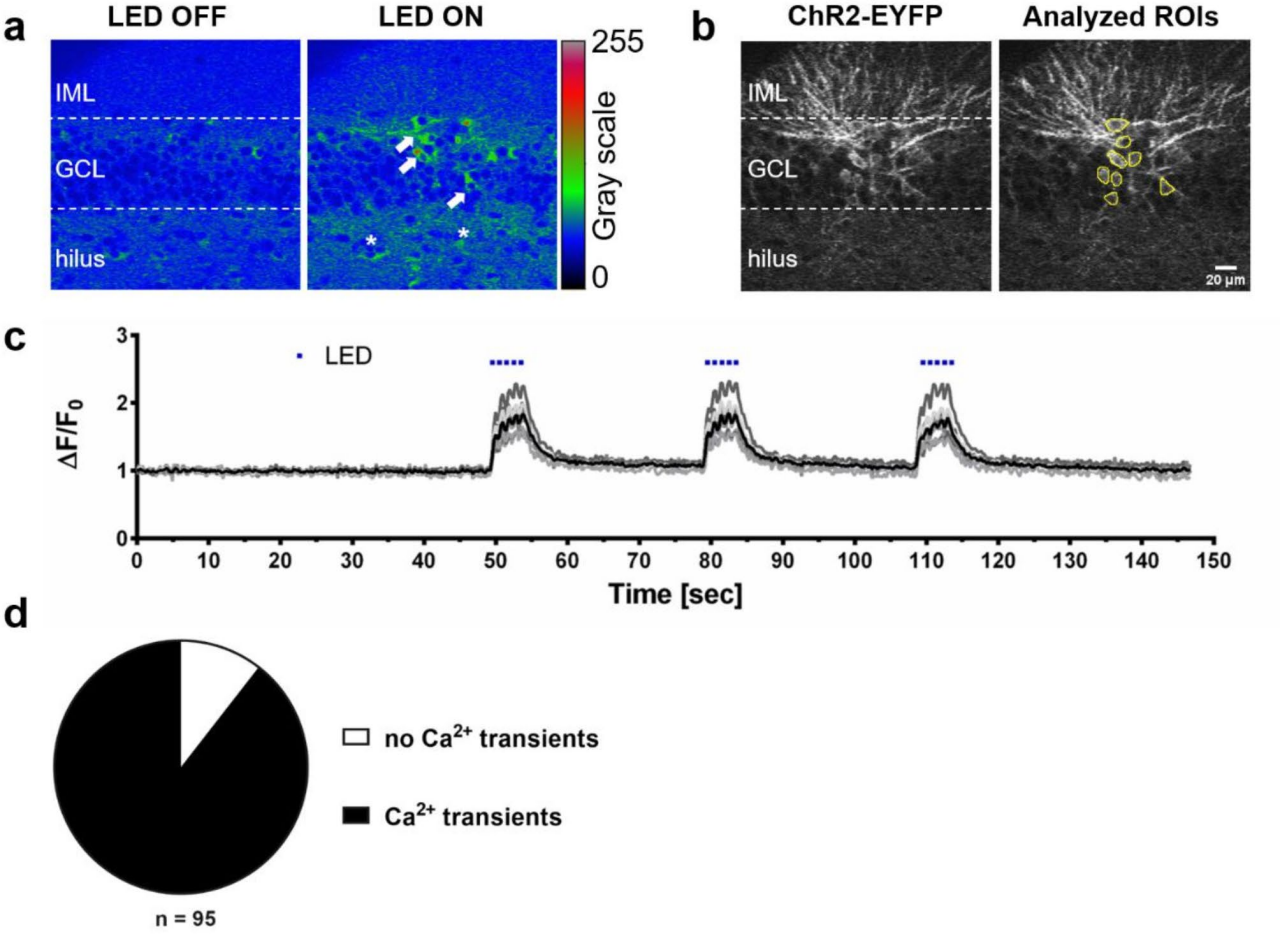
Optogenetic stimulation of ChR2-expressing GCs induces intracellular calcium transients. (**a**) A representative rainbow RGB image demonstrating the calcium response of DG neurons expressing the genetically encoded calcium indicator jRCaMP1b during 450 nm photostimulation. Under the LED OFF condition, spontaneous background activity was observed. Following stimulation with 450 nm light (LED ON) calcium signals were elicited in ChR2-EYFP-expressing GCs (white arrows) as well as some ChR2-negative hilar mossy cells (asterisks), indicating network activity in response to GC activation. (**b**) ChR2-transduced cells were visualized using the EYFP tag. All ChR2-expressing GCs within a given imaging frame were identified, regions of interest were assigned, and calcium changes within these regions of interest were analyzed over time. (**c**) Sample traces of normalized fluorescent changes showing calcium transients of 8 ChR2-expressing GCs in response to 3 trains of blue light stimulation with 5 × 500 ms light pulses. (**d**) The proportion of GCs exhibiting calcium transients was 89.5%, while 10.5% showed no response (*n* = 95). IML: inner molecular layer; GCL: granule cell layer. n represents number of cells.

For this series of experiments, the genetically encoded calcium indicator (GECI) jRCaMP1b was used to visualize calcium transients in ChR2-expressing GCs. This GECI has red-shifted excitation and emission spectra^30^ and is therefore particularly suitable for the combination with optogenetic approaches using the ChR2-H134R variant, as imaging of the calcium signal with a 561 nm laser does not cause ChR2 activation. OTCs received a local injection with ChR2-EYFP into the GCL and were then bulk-loaded with jRCaMP1b. This approach ensured a locally restricted expression of the ChR2-EYFP in GCs and a broad transduction of neurons with jRCaMP1b. Stimulation of OTCs with blue light was performed using a 450 nm LED module and recordings of calcium signals were performed from above using an upright confocal microscope detecting light emissions in the red. This approach revealed distinct calcium responses in ChR2 expressing GCs (Fig. 3a). Of note, the calcium signals showed simultaneous activation of a few hilar mossy cells (asterisks in Fig. 3a) in response to GC activation, suggesting again simultaneous discharge of many GCs presynaptic to these mossy cells. However, we did not specifically record nor quantify mossy cell responses, therefore further analyses will be necessary to shed light on specific network effects. The analysis of all ChR2-expressing neurons (Fig. 3b) within an imaging frame that were stimulated with three trains of 5 pulses (500 ms pulse length) at 1 Hz, showed calcium transients in response to every light pulse with similar responses elicited by each repetition of the stimulation train (Fig. 3c): 89.5% of ChR2-EYFP expressing GCs generated calcium transients, indicating the firing of APs, while 10.5% of GCs showed no substantial response to light (Fig. 3d).

### Physiological properties of ChR2-expressing GCs are indistinguishable from non-transduced controls

**Fig. 4:**
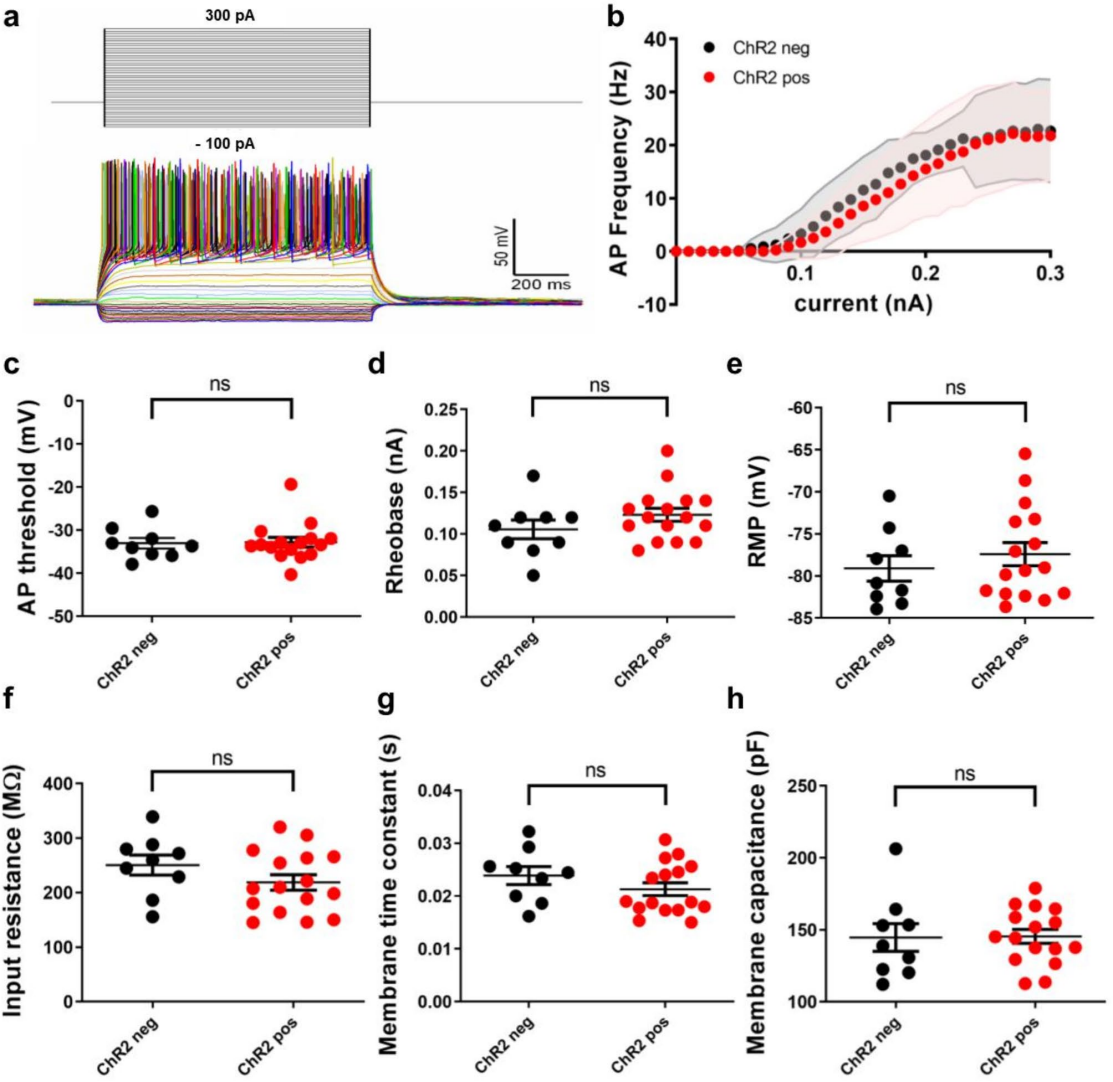
Physiological properties of ChR2-expressing GCs are indistinguishable from non-transduced controls. (**a**) Whole-cell patch-clamp recordings of dentate GCs were performed in current-clamp mode. Current steps ranging from − 100 pA to 300 pA, in 10 pA increments, were injected. Passive and active properties of ChR2-tdTomato positive and negative GCs from virus-injected cultures were compared. (**b**) The frequency of APs in response to current injection was similar between ChR2-positive (*n* = 16) and ChR2-negative GCs (*n* = 9). (**c**, **d**) There were no significant differences in the first AP threshold (Brunner-Munzel test, *P* = 0.82), nor the rheobase (*P* = 0.20) between the two cohorts. (**e**-**h**) We observed no significant differences in the resting membrane potential (*P* = 0.46), the input resistance (*P* = 0.17), the membrane time constant (*P* = 0.23), nor the membrane capacitance (*P* = 0.62) between ChR2-positive and ChR2-negative GCs. n represents number of cells. Bars represent means ± SEM.

Since the viral-vector based delivery and the long-term overexpression of the ChR2-protein could potentially influence electrophysiological properties of GCs^42,43^, we recorded basic physiological properties and firing characteristics of ChR2-transduced GCs and compared them with non-transduced GCs in virus-injected cultures. Both groups of GCs were subjected to electrical stimulation through square current pulses, with the injected current increasing in 10 pA increments with each iteration, in ACSF containing synaptic blockers (Fig. 4a). Intrinsic excitability was not altered: Neither the AP threshold potential of the very first elicited AP in each cell (ChR2-neg: -33.0 ± 1.2 mV; ChR2-pos: -32.8 ± 1.1 mV; Brunner-Munzel test, *P* = 0.82), nor the rheobase of that AP (ChR2-neg: 0.11 ± 0.01 nA; ChR2-pos: 0.12 ± 0.01 nA; *P* = 0.20) was different between neurons that were transduced with ChR2 and non-ChR2-expressing neurons. In addition, the increase in number of APs elicited by increasing current steps was very similar with or without ChR2 expression (Fig. 4b-d). Furthermore, we observed no significant differences in the resting membrane potential (ChR2-neg: -79.1 ± 1.5 mV; ChR2-pos: -77.4 ± 1.4 mV; *P* = 0.46), the input resistance (ChR2-neg: 250.5 ± 18.4 MΩ; ChR2-pos: 218.7 ± 14.2 MΩ; *P* = 0.17), the membrane time constant (ChR2-neg: 0.024 ± 0.002 s; ChR2-pos: 0.021 ± 0.001 s; *P* = 0.23), nor the membrane capacitance (ChR2-neg: 144.7 ± 9.7 pF; ChR2-pos: 145.5 ± 4.9 pF; *P* = 0.62) between the two cohorts (Fig. 4e-h). Overall, these data show that the incorporation of ChR2 into the cell membrane did not affect the intrinsic physiological properties of transduced GCs.

### Antioxidants protect OTCs from phototoxic damage during chronic photostimulation with blue (450 nm) and green (505 nm) light

**Fig. 5:**
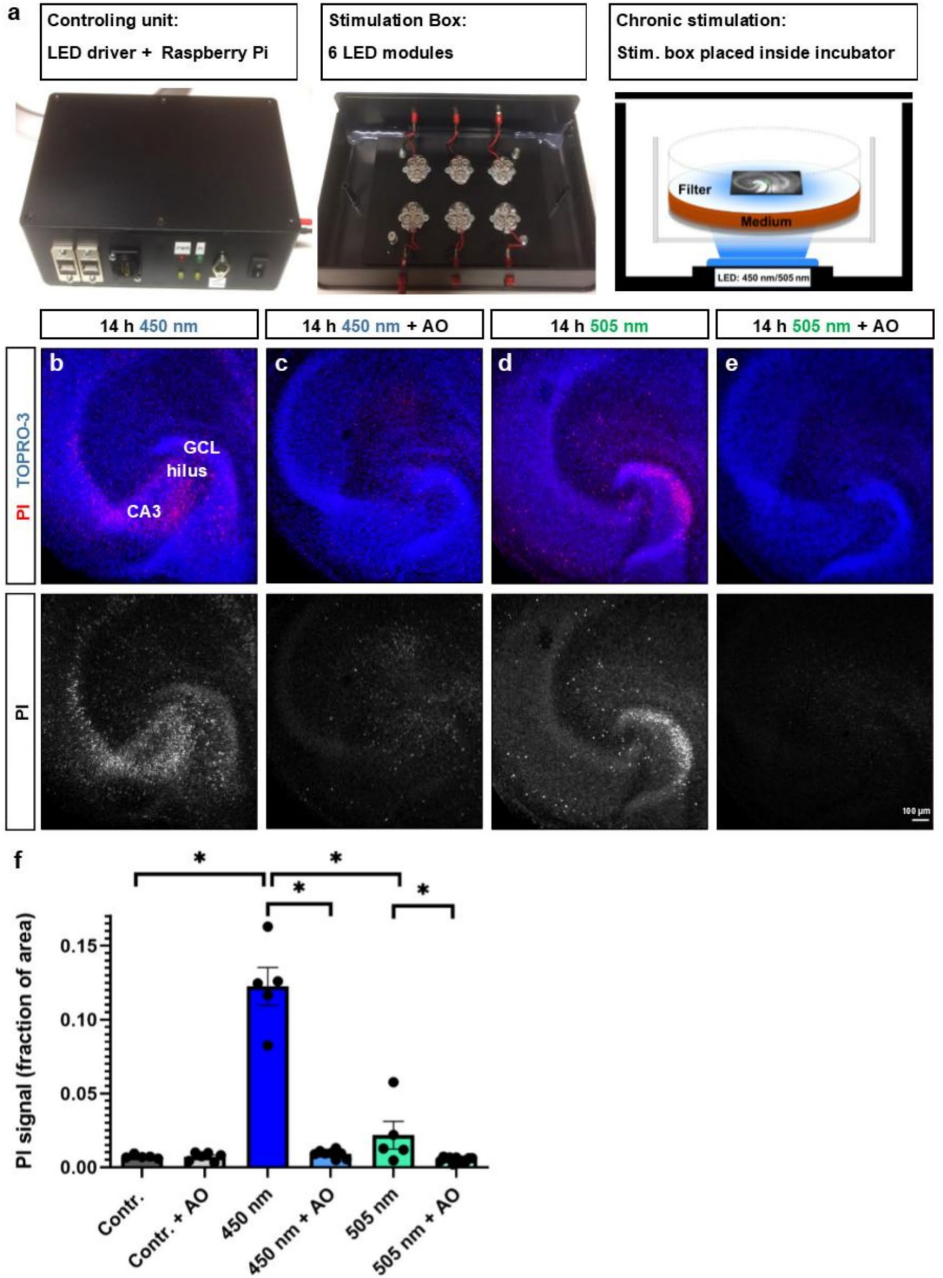
Antioxidants (AO) protect OTCs from phototoxic damage during chronic photostimulation with blue (450 nm) and green (505 nm) light. (**a**) Propidium iodide (PI) staining was used to detect phototoxic damage or cell death in OTCs after 14 h of strong photostimulation (100 pulses/minute at 12.5 Hz) using 450 nm or 505 nm LEDs. For the stimulation, cultures were placed inside a custom-built stimulation box that was controlled by a Raspberry Pi computer. Each individual membrane insert containing ChR2-transduced OTCs was illuminated with an LED module from below. (**b**, **d**) PI-positive cells were detected in the granule cell layer (GCL), hilus, and the CA areas of stimulated OTCs. (**c**, **e**) The addition of AO to the incubation medium dramatically reduced the amount of cell damage or death. (**f**) A quantification of the PI signal showed that cell damage was most pronounced in the 450 nm condition compared to the unstimulated control (two-way robust ANOVA with *post hoc* Brunner-Munzel tests, *P* = 0.020) as well as compared with cultures that were stimulated with 505 nm LEDs (*P* = 0.020). Furthermore, OTCs that were stimulated with either wavelength and treated with AO contained a significantly lower number of PI-positive cells compared with stimulated cultures that were not incubated with AO (450 nm vs. 450 nm + AO: *P* = 0.016; 505 nm vs. 505 nm + AO: *P* = 0.043). Contr.: *n* = 5; Contr. + AO: *n* = 6; 450 nm: *n* = 5; 450 nm + AO: *n* = 8; 505 nm: *n* = 5; 505 nm + AO: *n* = 11. n represents number of OTCs. Bars represent means ± SEM. *P < 0.05.

One of the advantages of using optogenetic stimulation in OTCs is the possibility to employ contact-free chronic neuronal stimulation over hours and days. Nevertheless, extended exposure to intense light may potentially induce cellular damage. In this context, we evaluated phototoxic damage caused by prolonged exposure to high-power LED light, considering cell viability. OTCs were incubated with propidium iodide (PI), a nucleic acid intercalating dye that is only permeant to damaged or dead cells^34^, directly after photostimulation using our custom stimulation boxes (Fig. 5a). Subjecting OTCs to chronic optical stimulation lasting 14 h (every minute one train of 100 pulses with 10 ms pulse length at a frequency of 12.5 Hz, i.e. a total of 84,000 light pulses) caused neuronal damage across the slice culture, predominantly in hippocampal areas CA1 and CA3 as well as the DG (Fig. 5b, d). We tested whether a cocktail of antioxidants (AO; i.e. glutathione, catalase, ascorbic acid, trolox, α-tocopherol, and superoxide dismutase)^44^ could mitigate these effects by adding these to the culture medium prior to illumination (Fig. 5c, e). It has been shown that the phototoxicity of light exposure depends on the wavelength of light employed, with shorter wavelengths having greater potential to harm compared to longer ones^45–47^. Hence, we outfitted our stimulation boxes with either 450 nm or 505 nm LEDs. OTCs were exposed to either blue (450 nm) or green (505 nm) light LEDs with light intensities adjusted to ~ 4.0 mW/mm^2^ or ~ 4.5 mW/mm^2^, respectively, over a 14-hour period (100 pulses with 10 ms pulse length at 12.5 Hz every minute). A two-way robust ANOVA showed significant differences in mean PI signal between the unstimulated control group (0.007 ± 0.001) and OTCs stimulated with 450 nm LEDs (0.122 ± 0.01) and 505 nm LEDs (0.022 ± 0.01; global stimulation effect: WTS = 83.72, *P* < 0.001). Pairwise comparisons using *post-hoc* Brunner-Munzel tests showed significant differences between control and 450 nm (*P* = 0.020) as well as 450 nm and 505 nm stimulation (*P* = 0.020; Fig. 5f). Furthermore, stimulated OTCs treated with AO contained significantly less PI signal compared to untreated stimulated cultures (global AO effect: WTS = 66.26, *P* < 0.001). The effect of stimulation depended on AO treatment (stimulation x AO interaction: WTS = 79.89, *P* < 0.001) in both, the 450 nm condition (450 nm: 0.122 ± 0.01; 450 nm + AO: 0.009 ± 0.00; *P* = 0.016) and the 505 nm condition (505 nm: 0.022 ± 0.01; 505 nm + AO: 0.005 ± 0.001; *P* = 0.043; Fig. 5f). These tests showed that relatively strong stimulation was damaging to OTCs, especially when 450 nm light was used. However, in OTCs that were incubated with AO during light exposure, the number of PI-positive cells was drastically reduced. We also assessed the effect of a chronic but more moderate light stimulation protocol and administered five pulses with 10 ms pulse length every hour at a frequency of 12.5 Hz over four days using 450 nm LEDs at a light intensity of ~ 4.0 mW/mm^2^. The PI staining showed no cell death in this case (data not shown), indicating that this chronic stimulation protocol does not lead to phototoxic cell damage. In sum, the use of AO can protect OTCs from neurotoxic damage. However, based on our data, it appears to be important to control for phototoxic damage if chronic stimulation of OTCs is performed using high frequency stimulation protocols.

### Denervation-induced spine loss is mitigated by chronic light-activation of denervated GCs

**Fig. 6:**
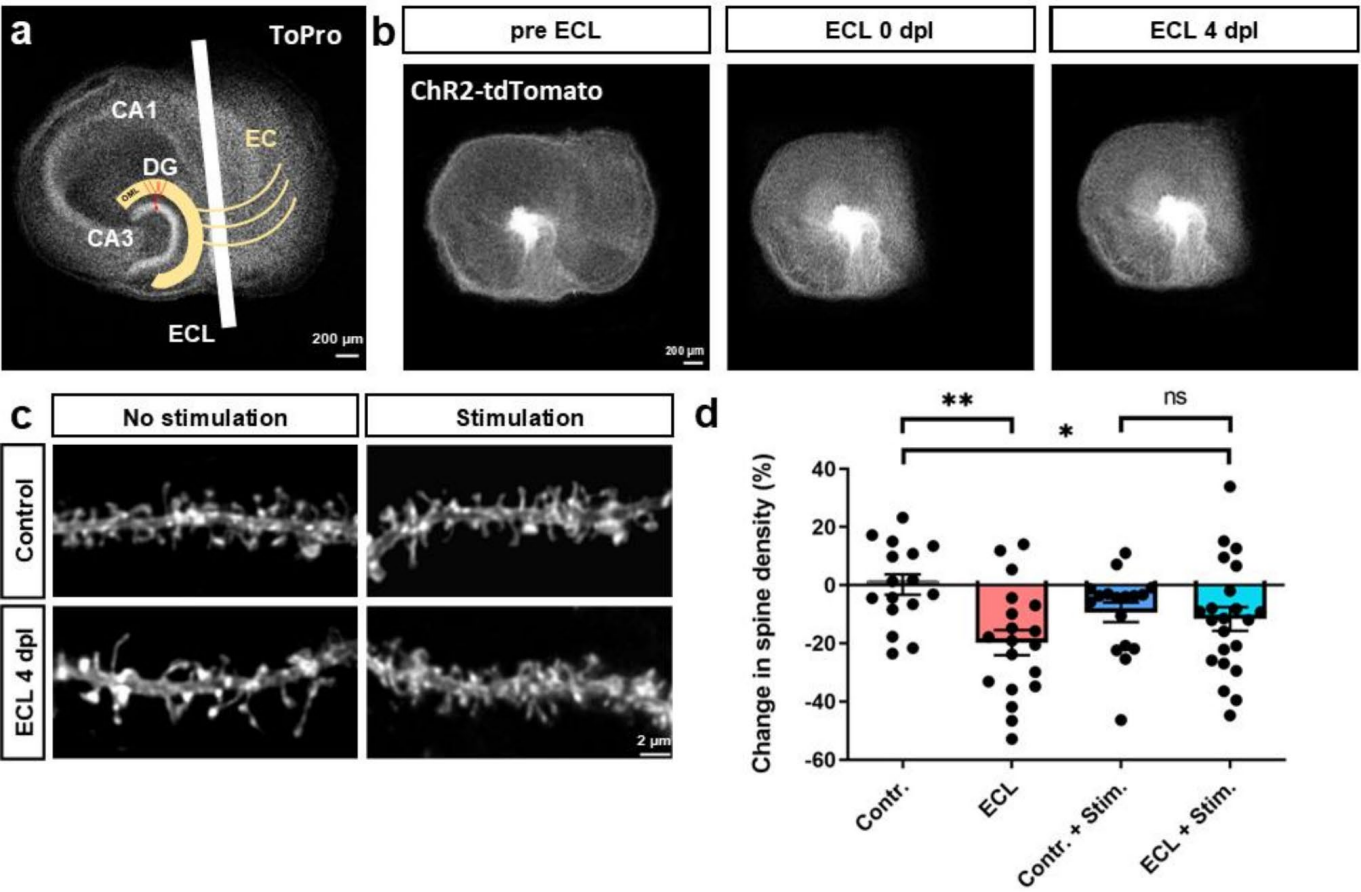
Denervation-induced spine loss is mitigated by chronic light-activation of denervated GCs. (**a**) Semi-schematic diagram illustrating the entorhinal cortex lesion (ECL) in vitro model. The perforant pathway from the entorhinal cortex (EC) to the dentate gyrus (DG) is cut under optical control using a sterile blade and the EC is removed from the dish. In response to entorhinal denervation, GCs lose a significant portion of their dendritic spines. (**b**) Example of an OTC transduced with ChR2-tdTomato before ECL, immediately following ECL, and 4 days post lesion (dpl). (**c**) To test whether optogenetic activation of GCs can protect denervated GCs from spine loss, OTCs were subjected to four different conditions. One set of denervated and control cultures was not illuminated, whereas the other set of denervated and control cultures was chronically illuminated with 450 nm LEDs over four days using a low-frequency stimulation protocol (5 pulses with a pulse length of 10 ms at 12.5 Hz every hour). (**d**) A quantification of changes in spine density between day 0 (pre ECL) and 4 dpl showed a significant difference between non-illuminated control cultures and OTCs that have been denervated (two-way robust ANOVA with *post hoc* Brunner-Munzel tests, *P* = 0.002; Control: *n* = 16; ECL: *n* = 19), exhibiting the characteristic spine loss at 4 dpl. There was no significant difference between non-denervated control cultures that were not stimulated and those that were stimulated (*n* = 17) nor between denervated cultures that were not stimulated and those that were stimulated (*n* = 22). Importantly, there was also no significant difference between LED-stimulated OTCs that were non-denervated and those that had been denervated. These findings suggest that the effect of denervation was attenuated in cultures that were illuminated. Indeed, the effect of ECL depended on LED stimulation (ECL x stimulation interaction: WTS: 5.30; *P* = 0.024). Nevertheless, denervated OTCs that were stimulated were still significantly different from the non-illuminated, non-denervated control (*post hoc* Brunner-Munzel test, *P* = 0.037). n represents number of dendritic segments. Bars represent means ± SEM. *P < 0.05; **P < 0.01.

Finally, we performed a proof-of-principle experiment and used chronic low-frequency light activation of dentate GCs over four days to directly activate GCs with light and to compensate for the loss of excitatory afferent input to GCs after entorhinal denervation. As has been reported previously, entorhinal denervation in vitro (Fig. 6a, b) results in the loss of all excitatory entorhinal afferents to the distal dendrites of GCs^23,31^. Following this trauma, GCs stop firing spontaneous APs^48^ and remodel their dendritic tree^32,49^. By four days post lesion (dpl), they lose a significant portion of their dendritic spines^32^.

Since the above changes are associated with a reduction in GC activity, we speculated that direct activation of GCs with light could compensate for the loss of excitatory input. Therefore, we transduced GCs with ChR2-tdTomato and chronically activated ChR2-expressing GCs using a low frequency protocol (5 pulses with 10 ms pulse length at 12.5 Hz every hour) with an AP frequency in their physiological activity range^50^. TdTomato-fluorescence was used to visualize dendritic segments and their spines (Fig. 6c) and the effect of denervation on dendritic spine density was used as a structural read-out. The quantification of changes in spine density between day 0 (pre ECL) and 4 dpl shows a significant difference between non-illuminated non-denervated control cultures (0.18 ± 3.51%) and non-illuminated denervated cultures (-19.82 ± 4.33%; two-way robust ANOVA: Global ECL effect: WTS = 8.37, *P* = 0.005, followed by *post hoc* Brunner-Munzel tests, *P* = 0.002; Fig. 6d). There was no significant difference between non-denervated control cultures that were not stimulated and those that were stimulated, nor between denervated cultures that were not stimulated and those that were stimulated. Importantly, there was also no significant difference between LED-stimulated OTCs that were non-denervated and those that had been denervated. These findings suggest that the effect of denervation was attenuated in cultures that were illuminated. Indeed, the effect of ECL depended on LED stimulation (ECL x stimulation interaction: WTS: 5.30; *P* = 0.024). Nevertheless, denervated OTCs that were stimulated (-11.63 ± 4.10%) were still significantly different from the non-illuminated, non-denervated control (*post hoc* Brunner-Munzel test, *P* = 0.037).

## Discussion

In this study, we established optogenetics in complex entorhino-hippocampal OTCs to directly activate mouse GCs with light. We report robust GC activation in vitro and, furthermore, demonstrate that chronic direct activation of GCs by light illumination mitigates denervation-induced structural changes of GCs. Our findings can be summarized as follows: (1) Local injections of adeno-associated viruses were employed to selectively transduce GCs in OTCs with optogenetic constructs. (2) Patch-clamp recordings demonstrated that basic electrophysiological parameters of transduced GCs were not altered and that these cells were excitable with light. (3) Calcium-imaging showed that ~90% of all transduced GCs generated robust calcium transients in response to photostimulation. (4) Efficiency of activation depended on light intensity and expression level of ChR2. (5) Antioxidants prevented phototoxic damage by chronic light illumination. (6) Finally, chronic activation of GCs in their physiological activity range mitigated spine loss induced by entorhinal denervation. In sum, we report here the effects of acute and chronic optogenetic activation of mouse GCs in OTCs under non-denervated control and under denervation conditions.

### Local injections allow for the direct comparison of transduced and non-transduced GCs in the same cultures

For the transduction of dentate GCs with channelrhodopsins or genetic calcium indicators, adeno-associated viruses were used as vectors and injected into or close to the GCL^11,23,26^. This resulted in the transduction of a subset of ~10–50 GCs, which were readily identified in the confocal microscope (Fig. 1). This limited transduction was ideal for a cellular analysis, since transduced cells are embedded in a larger network of wildtype neurons. Network effects, which may occur if all or most neurons of a network are genetically altered, e.g. after bulk loading of cultures with viruses, are unlikely to confound results. Furthermore, transduced GCs can be directly compared with non-transduced GCs in the same cultures. These non-transduced GCs are ideal controls, since they were treated in exactly the same way as the transduced GCs - with the exception of the viral transduction - and also received the same network activity. We exploited this situation in our study and compared the basic electrophysiological parameters of ChR2-tdTomato expressing neurons with non-transduced neurons in virus-injected cultures. Under these conditions, no significant differences were observed between the two groups, demonstrating that the viral transduction with ChR2-tdTomato did not lead to a major change in the electrophysiological properties of the transduced cells (Fig. 4).

### Action potential induction in transduced GCs depends on light intensity and expression level of ChR2

Next, we verified that ChR2-transduced cells were activated by light using patch-clamp recordings. Since the activation of channelrhodopsins depends on the biophysical properties of the channelrhodopsin^29^, we determined parameters to robustly activate identified GCs in the cultures. Furthermore, we observed that expression levels of ChR2 - accessed by fluorescence intensity measurements - varied between individual GCs. The number of virus particles taken up by a single cell and/or the amount of protein synthesized by a cell may readily explain individual differences in the response of GCs to light stimulation.

Regarding the parameters to reliably stimulate GCs in OTCs, we tested two different wavelengths (450 nm and 505 nm) at increasing light intensities. These wavelengths have been applied previously in published work using the ChR2 employed in our study^27,29^. The genetically engineered ChR2 that contains the H134R point mutation produces larger photocurrents but has relatively slow channel kinetics compared with the wildtype^27,29^. Our analyses revealed that the probability of GCs firing APs upon photostimulation depended on the light intensity. For both blue and green light conditions, we found that there were essentially two GC populations with one large cohort of neurons firing APs in response to relatively low light intensities (i.e. <2 mW/mm^2^) and others spiking only when stimulated with higher light intensities. We hypothesized that the differences in response rate may be related to the expression level of ChR2, hence we analyzed the ChR2-tdTomato signal in individual GC somata. Our results show that GCs that were activated by low light intensities exhibited relatively high ChR2 expression levels on average, while GCs that responded only to higher light intensities and those that failed to spike at all had relatively low ChR2-expression (Fig. 2).

Since fewer channels result in a smaller cation-influx, we expected and confirmed a reduced excitability of weakly expressing cells compared to strongly expressing cells. Higher light intensities were needed, possibly saturating the channelrhodopsins, to elicit APs in these cells. Even though higher expression levels in individual GCs are preferable to ensure reliable firing in response to light stimulation, caution must be employed to keep overall transduction levels moderate, as very high numbers of transduced cells may lead to cytotoxicity^42^.

### Calcium-imaging demonstrates robust calcium transients in activated GCs

Using a genetic calcium indicator, we recorded calcium transients in response to light stimulation of all ChR2-expressing GCs within a given culture. We found that ~90% of GCs co-expressing ChR2 and the calcium indicator jRCaMP1b exhibited calcium transients (Fig. 3). This finding is in line with our patch-clamp data and it underscored our observation that the excitability of neurons depends on their individual ChR2 expression level.

The combination of calcium imaging, optogenetics, and live time-lapse imaging appears to be an attractive technique for an all-optical study of neuronal structure and function in OTCs. In contrast to patch-clamp recordings, which are limited to minutes or at best some hours of one or a few cells simultaneously, calcium imaging can be employed repeatedly over extended time periods and in large cell populations. Contrariwise, recordings with multi-electrode arrays do not reveal structural cellular identities. In addition, recently developed open source software packages^51^ provide a data processing pipeline for automated analysis of calcium imaging data, which is ideal for large datasets. Coupling this with correlation analysis will make it possible to not only study single cells, but also their interactions in the network.

### Antioxidants (AO) prevent phototoxic damage following chronic stimulation of OTCs with light

Intensive illumination with strong light may have phototoxic effects. We tested for potential harmful effects of blue and green light by subjecting OTCs to strong photostimulation with either 450 nm or 505 nm LEDs for 14 h. Using PI staining, indications for nuclear damage and cell death were observed in all regions and subfields of the hippocampus, including the dentate GCL. In previous studies in which chronic photostimulation was applied in OTCs, researchers have used AO, presumably to protect cells from phototoxic damage^44,52^. We adapted their protocol and found that the addition of AO to the incubation medium protected chronically stimulated OTCs from cell death (Fig. 5). Nevertheless, phototoxic damage remains a confounding factor, especially if stronger illumination protocols are used.

Light-induced phototoxicity may be a specific problem of cell culture preparations. Previous reports have suggested that components of the cell culture medium, such as tryptophan and riboflavins, which are capable of light absorption, generate radicals, thereby contributing to phototoxicity^53,54^. Choosing genetically engineered channelrhodopsins that open at longer wavelengths or lower light intensities^55,56^, highly focused light illumination, or the use of AO^44,52^ may help to avoid this problem.

### Denervation-induced spine loss is mitigated by chronic light-activation of denervated GCs

In previous studies, we have carried out transections of the perforant pathway in OTCs under visual control^3,31,32^. We reported that denervated GCs react to the loss of the majority of their excitatory inputs with a decrease in spine density, which reaches its maximum around four days post lesion^32^. Loss of spines after denervation can be reduced by pharmacological AMPA-R activation^57^, suggesting that the lack of synaptic activation causes retraction of spines following denervation. We wondered whether some degree of direct postsynaptic activation might prevent removal of denervated spines independent of synaptic activation. It has been reported that GCs decrease their spontaneous firing activity following entorhinal denervation^58^. Thus, backpropagating APs entering their dendrites may be less frequent. We hypothesized that a direct chronic induction of AP firing in GCs in the absence of entorhinal input could rescue their spines. Indeed, chronic light-activation of denervated GCs at the physiological activity range of GCs^50^ resulted in a mitigation of spine loss after denervation, compared to their corresponding control counterpart (Fig. 6). This rescue effect might be caused by the depolarization due to opening of ChR2 present in spines, somehow equivalent to AMPAR-induced postsynaptic potentials. However, McKinney and co-authors (1999), suggested a trophic effect of AMPAR activation, independent of calcium influx and large depolarizations. Our work demonstrated the general feasibility of the optogenetic approach to protect denervated GCs from structural changes and will facilitate further studies to reveal the ideal stimulation protocol and underlying mechanisms for a long-term protection of GCs and other neurons following denervation.

## Data Availability

The datasets generated during and/or analyzed during the current study are available from the corresponding author on reasonable request.
